# Sequences Encoding a Novel Toursvirus Identified from Southern and Northern Corn Rootworms (Coleoptera: Chrysomelidae)

**DOI:** 10.3390/v14020397

**Published:** 2022-02-15

**Authors:** Sijun Liu, Thomas W. Sappington, Brad S. Coates, Bryony C. Bonning

**Affiliations:** 1 Department of Entomology, Iowa State University, Ames, IA 50011, USA; sjliu42@gmail.com; 2 Corn Insects and Crop Genetics Research Unit, USDA-ARS, Ames, IA 50011, USA; Tom.Sappington@usda.gov (T.W.S.); Brad.Coates@usda.gov (B.S.C.); 3 Department of Entomology and Nematology, University of Florida, Gainesville, FL 32611, USA

**Keywords:** *Ascoviridae*, toursvirust, beetle, corn rootworm, *Diabrotica* spp.

## Abstract

Sequences derived from a novel toursvirus were identified from pooled genomic short read data from U.S. populations of southern corn rootworm (SCR, *Diabrotica undecimpunctata howardi* Barber) and northern corn rootworm (NCR, *Diabrotica barberi* Smith & Lawrence). Most viral sequences were identified from the SCR genomic dataset. As proteins encoded by toursvirus sequences from SCR and NCR were almost identical, the contig sets from SCR and NCR were combined to generate 26 contigs. A total of 108,176 bp were assembled from these contigs, with 120 putative toursviral ORFs identified indicating that most of the viral genome had been recovered. These ORFs included all 40 genes that are common to members of the *Ascoviridae*. Two genes typically present in *Ascoviridae* (*ATP binding cassette transport system permeases* and *Baculovirus repeated open reading frame*), were not detected. There was evidence for transposon insertion in viral sequences at different sites in the two host species. Phylogenetic analyses based on a concatenated set of 45 translated protein sequences clustered toursviruses into a distinct clade. Based on the combined evidence, we propose taxonomic separation of toursviruses from *Ascoviridae*.

## 1. Introduction

A complex of four corn rootworm species and subspecies are native to North America; western corn rootworm (WCR), *Diabrotica virgifera virgifera* (LeConte), southern corn rootworm (SCR), *Diabrotica undecimpunctata howardi* (Barber), Mexican corn rootworm, *Diabrotica virgifera zeae* (Krysan & Smith), and northern corn rootworm (NCR), *Diabrotica barberi* (Smith & Lawrence) [1]. These species pose significant threats to maize production in the United States, and cause estimated annual losses of $2 billion in cumulative yield and control costs [2]. Efforts to manage damage to maize crops have been hampered by adaptations to crop rotation in WCR [3] and NCR [4] populations. Furthermore, WCR has developed resistance to multiple insecticide chemistries [5]. More recently resistance to transgenic maize hybrids that express *Bacillus thuringiensis* (Bt) pesticidal proteins has been documented in WCR [6,7,8] and NCR [9]. Consequently, a more integrated approach to corn rootworm management has been proposed [10].

The use of viruses for suppression of corn rootworm populations provides an alternative strategy within an integrated approach for crop protection. The practical utility of viral-based biological control is exemplified by use of a nudivirus for control of the Asiatic rhinoceros beetle, *Oryctes rhinoceros* (L.) [11,12]. While microscopic observations have suggested the presence of DNA viruses in *Diabrotica* spp. including in SCR [13,14], none were defined as ascovirus-like nor were any associated pathologies observed [15,16].

*Ascoviridae* is a family comprised of viruses with circular, double-stranded DNA genomes that fall into one of two genera, *Ascovirus* and *Toursvirus* [17]. The genomes of *Toursvirus* are shorter (120–143 kbp) than those of *Ascovirus* (157–200 kbp). Eleven full length genome sequences of viruses belonging to five ascovirus species have been reported, with 119–194 predicted ORFs of which 40 are shared among them (Table 1). Phylogenetic analyses indicate that ascoviruses evolved from an ancestral iridovirus [18].

Ascoviruses in the genus *Ascovirus* primarily infect lepidopteran larvae in the family *Noctuidae* and are vectored during oviposition by parasitoid wasps. Importantly for their potential use as biocontrol agents, ascoviruses cause chronic and fatal disease in their larval lepidopteran hosts [28]. An ascovirus identified from a parasitoid wasp of lepidopterans, *Diadromus pulchellus,* originally named *Diadromus pulchellus ascovirus 4a* (DpAV) [29] was renamed *Diadromus pulchellus toursvirus 1a* (DpTV1a) on establishment of the *Toursvirus* genus [17]. This sole member of the genus *Toursvirus* replicates extensively in lepidopteran hosts and in the primary parasitoid vector *D. pulchellus* to a limited extent, with the viral genome existing in the wasp as unintegrated DNA. The first non-lepidopteran ascovirus, Dasineura jujubifolia toursvirus 2a (DjTV2a) was isolated from a dipteran, and is closely related to DpAV1a. However, no obvious disease symptoms were observed in DjTV2-infected insects [26].

To assess the potential for virus-based suppression of *Diabrotica* spp., we examined the associated virome drawing on both genomic and transcriptomic sequence data [30]. From this we found evidence for a diverse set of corn rootworm viruses. Findings include sequences derived from three novel small RNA viruses from WCR transcripts [31,32,33] and two from SCR transcripts; and DNA sequences of two novel nudiviruses derived from the SCR and WCR genomes [34]. Here we report a novel toursvirus, with the genome sequence assembled from short sequence reads derived from both SCR and NCR DNA extracts. This is the first putative member of *Ascoviridae* isolated from Coleoptera.

## 2. Materials and Methods

### 2.1. SCR and NCR Sample Collection and DNA Isolation

Adult SCR (*n* = 50) were collected from Ames, Iowa. Methods for SCR total genomic DNA isolation followed those previously described [34]. NCR samples were collected from a grower’s field near Monmouth, IL in late July of 2012. All samples were flash frozen in liquid nitrogen and stored at −80 °C. The NCR sample was comprised of 35 males and 36 females (*n* = 71) that were pooled. The sample was ground to a powder in liquid nitrogen. DNA was extracted from 3.0 mg of ground NCR material using the Qiagen DNeasy Blood and Tissue Extraction kit (Qiagen, Germantown, MD, USA), with modifications as described [35].

### 2.2. Sequencing Library Preparation and Illumina Sequencing

Purified DNA was submitted to the Iowa State University DNA Facility (Ames, IA, USA). Genomic DNA was size selected and used to generate ~500 bp insert libraries using Illumina TruSeq v2 Library Construction Kits (Illumina, San Diego, CA, USA). Single-end 100-bp Illumina HiSeq2500 reads were generated, with SCR and NCR libraries run in separate lanes. Data were received in raw fastq format and were submitted to the National Center for Biotechnology Information (NCBI) Short Read Archive (SRR13364002 for SCR, SRR13363759 for NCR). Raw reads were trimmed to remove low-quality nucleotides as previously described [35] prior to further use in this study.

### 2.3. Sequence Assembly and Annotation

SCR and NCR sequence data were assembled as described previously [34,36]. Briefly, the trimmed DNA sequence reads from SCR and NCR were assembled using Trinity (v2.6.6) [37], followed by reduction in sequence redundancy using CAP3 [38]. Contigs over 450 bp were selected and used for viral sequence annotation. The selected contigs were first used as queries against a local insect DNA viral protein sequence database with the BLASTx algorithm [39] embedded in Bioedit v.7.2 [40] (https://bioedit.software.informer.com/; accessed on 15 November 2020). BLASTx results were filtered for *E* values ≤ 0.0001. These contigs were further used as queries against the NCBI nr database with the BLASTx algorithm. Contigs with “hits” to viral sequences were sorted based on putative virus species of the hit. DNA fragments with alignments to ascovirus and iridovirus sequences were selected for further analyses. As the putative toursvirus sequences derived from NCR and SCR encoded proteins had 100% identity, the two sets of contigs were merged. Potential coding sequences (CDS) (≥50 aa) were translated using SnapGene Viewer (SnapGene software—Insightful Science; available at snapgene.com; accessed 2-1-21). Individual protein translations were then aligned to those in the NCBI nr protein database using BLASTp as described previously [34].

### 2.4. Viral Sequence Analysis

Further sequence alignments and other manipulations were performed using Bioedit [40]. Details of SCR and NCR viral protein translation, protein molecular mass, location of an ORF within the genome fragments and related information were generated by SnapGene Viewer (GSL BioTech LLC, San Diego, CA, USA). The ORF and other features in SCR and NCR viral DNA fragments were visualized using maps generated by SnapGene Viewer. Methods for viral sequence mapping with the sequencing reads have been previously described [34,36]. The protein sequences encoded by 45 genes derived from a total of 26 viral genomes were selected on the basis of the BLAST analysis, for phylogenetic analysis (Table S1). Phylogenetic tree construction was performed with PyloSuite (v1.2.2) [41]. IQ-TREE methods were used to build the phylogenetic tree [42] including Edge-lined partition models for 5000 ultrafast bootstraps [43,44] and the Shimodaira–Hasegawa–like approximate likelihood-ratio test [45]. The resulting phylogeny was viewed using FigTree (v1.4.4) (http://tree.bio.ed.ac.uk/software/figtree/; accessed on 3 June 2021).

### 2.5. Analysis of Similairy between Toursvirus, Ascovirus and Other Invertebrate DNA Viruses

To infer phylogenetic relationships between toursviruses and related DNA viruses, the putative protein sequences of DpTV1a (119 protein sequences) and DjTV3a (141 protein sequences) were aligned to the NCBI nr database with the BLASTp algorithm. The species associated with the 10 most similar proteins were extracted and ranked from most- (1) to least- (10) similar.

## 3. Results

### 3.1. Novel Toursvirus-like Sequences Identified from SCR and NCR

Processing of short read genome sequence data from the SCR and NCR samples generated 118.5 and 9.7 million trimmed reads, respectively. Subsequent assemblies for SCR and NCR read data yielded 1,604,773 and 26,984 contigs (≥200 nt), respectively. Results from BLASTx searches against our local insect DNA viral protein sequence database showed “hits” among contigs for SCR samples with a previously identified novel nudivirus (*Diabrotica undecimpunctata howardi nudivirus*) [34]. Additionally, 28 unique DNA fragments, ranging from 469–19,547 bp from the SCR assembly showed significant identity with toursviruses. These putative novel toursvirus-like DNA fragments predicted a cumulative total of 115 protein coding sequences (CDS ≥ 50 aa). Similarly, BLASTx analysis of assembled NCR contigs revealed 42 unique DNA fragments ranging from 516–8299 bp, that showed “hits” to toursviral DNA accessions. Putative CDS translations for these NCR contigs predicted 117 putative viral protein coding genes.

### 3.2. Toursvirus Sequences Identified from SCR and NCR Derived from the Same Virus

**Figure 1:**
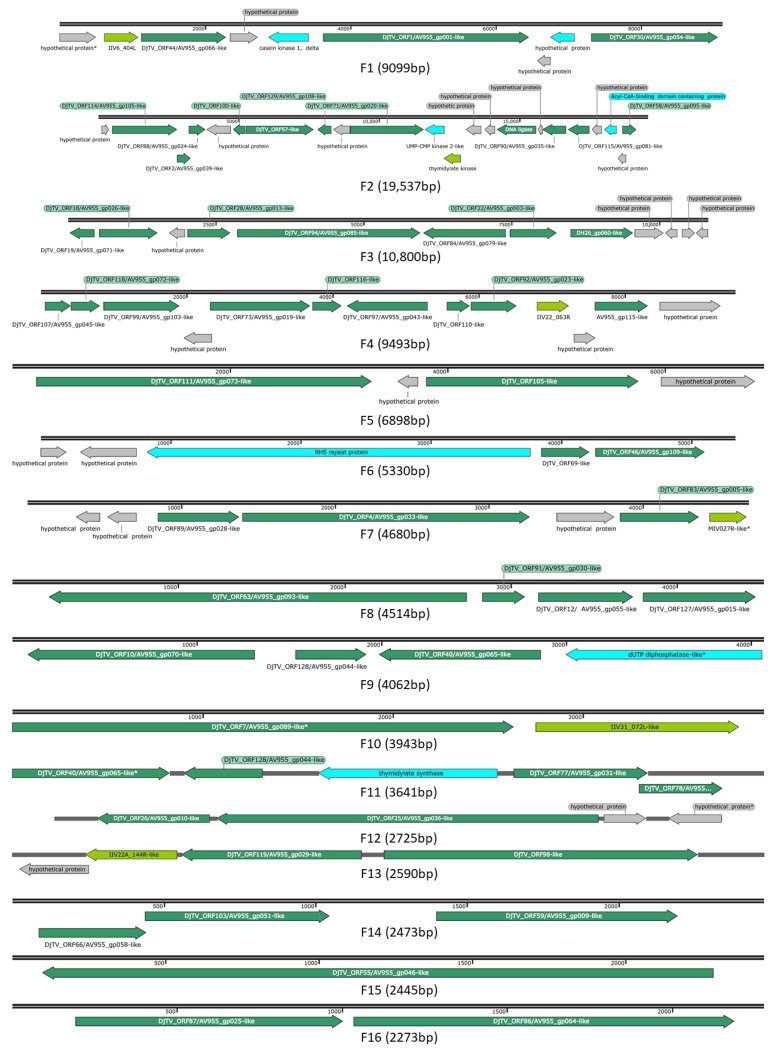

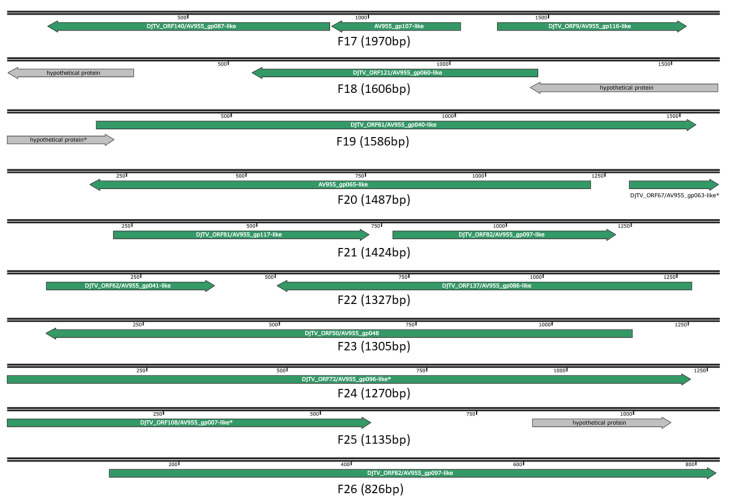
Map of the 26 DiTV3a genomic fragments. Fragments derived from both SCR and NCR were combined. Arrows indicate ORFs and ORF orientation. Dark green, ORFs that hit toursvirus genes (DjTV2a/DpTV1a); light green, similar to other viral genes; blue, ORFs that hit non-viral genes; grey, unknown ORFs. *, partial sequence. The fragments identified from SCR and NCR isolates are provided in Figures S1 and S2.

Annotation of CDS translations from the SCR and NCR contigs with initial putative BLASTx “hits” to toursvirus-like accessions by a secondary BLASTp query against the NCBI nr database further indicated similarities to toursviruses. Specifically, toursvirus protein accessions were the top BLASTp matches for our CDS translations from putative virus-derived contigs of SCR and NCR (Table S2). Due to the 100% amino acid identity between putative CDS translations from SCR and NCR identified by interspecific BLASTp alignment, these annotations were merged across SCR and NCR contigs (results not shown). This showed that, although the toursviral-like contigs varied in size, the order and orientation of the 120 CDS were conserved between the 28 and 42 genomic fragments from SCR (Figure S1) and NCR (Figure S2). As these data indicated that the toursvirus isolates from SCR and NCR derive from the same or similar viruses, the toursviral sequence fragments from SCR and NCR were merged to generate 26 unique toursviral consensus sequence fragments (F1-F26) for further analysis (Figure 1; Table 1 and Table S2). As the virus was identified from two different *Diabrotica* species, this novel toursvirus is named Diabrotica toursvirus 3a (DiTV3a).

### 3.3. Annotation of the Novel Toursvirus Sequences

The 26 toursvirus genome sequence fragments totaled 108,176 bp, nearly 10 kbp less than that of the DpTV1a genome, the shorter genome of the two toursviruses characterized to date (Table 1). This suggests that some of the genome sequence of the new toursvirus may not have been recovered from the SCR and NCR samples. The C+G content of the virus genome is 30% (Table 2), which is less than that of the two known toursviruses (Table 1). Mapping DNA sequence reads to the 26 DiTV3a genome fragments showed nucleotide coverages of ~52-fold and ~16-fold for SCR and NCR, respectively. The lower coverage from NCR may partially account for the shorter contig lengths in the assembly identified as toursvirus-like when compared to those in the SCR sample (Figures S1 and S2). One hundred and seven of the 120 putative ORFs (≥50 aa) identified in the 26 fragments of DiTV3a (Table 3 and Table S2) were full length based on comparisons to those from other toursviruses (Table S2), with 13 ORFs encoding putative partial protein sequences. Fifty nine percent of the 120 putative ORFS (71 ORFs) have similarity to proteins encoded by known toursviruses (DpTV1a and DjTV2a), while 25% (30 ORFs) lacked similarity to any protein sequences in GenBank (Table 3). The organization of ORFs in the toursviral DNA fragments is shown in Figure 1, and the corresponding ORFs found in the SCR and NCR are presented in Figures S1 and S2, respectively.

### 3.4. Analysis of the Putative DiTV3a Genes

Annotations assigned to the accession of top BLASTp “hit” were used to attribute potential function of putative DiTV3a ORFs (Table S2). The ORFs with similarity to known toursviruses are indicated in Figure 1. About 70% of the putative DiTV3a ORFs returned significant hits (*E* values < 0.001) to proteins in the NCBI nr database, with 51 and 20 of the top “hits” from DjAV2a (and DpTV1a, respectively (Table S2). Best matches were also predicted to viral genes from iridoviruses (5 hits), a mimivirus (*Acanthamoeba polyphaga mimivirus*), and a poxvirus (*Fowlpox virus*). Seven BLASTp “hits” were from non-viral proteins (bacterial, insect, a protozoan, and a nematode protein), and the remaining 30 putative DiTV3a proteins returned no hits (listed as “hypothetical proteins” in Table S2).

The sequences of these dsDNA viruses were similar to toursviral, ascoviral and iridoviral genes [25,26]. These genes were grouped into 7 functional categories based on their putative biological functions derived from gene annotations from related viruses (Table 3). Fifty nine percent of the 120 putative ORFS (71 ORFs) have similarity to proteins encoded by known toursviruses (DpTV1a and DjTV2a), while 25% (30 ORFs) lacked similarity to any protein sequences in GenBank (Table 3). From the 120 putative ORFs of DiTV3a, 40 are shared among known members of the family *Ascoviridae* (indicated in bold in Table 3 and Table S2). The presence of all 40 of these shared genes along with the number of putative ORFs identified relative to those of other ascoviruses (119 in DpTV1a and 141 in DjTV2a; Table 1), suggests that the vast majority of DiTV3a genes were recovered from the assembly of DNA sequencing reads. Notably, two types of genes commonly found in toursviruses and ascoviruses, *ATP binding cassette (ABC) transport system permeases* and *Baculovirus repeated open reading frame* (*bro*) [46] were not present in the recovered DiTV3a genomes.

### 3.5. Putative DiTV3a Genes Associated with Retrotransposon Elements

**Figure 2:**
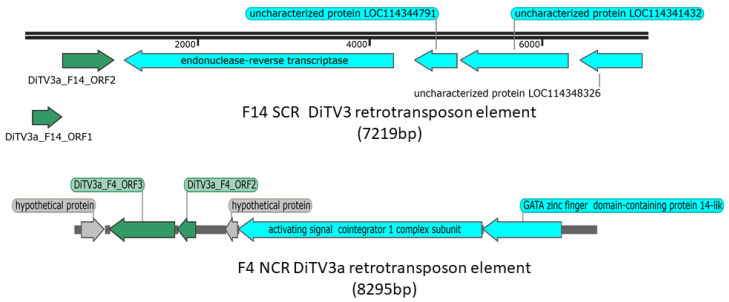
DiTV3a fragments fused to retrotransposon elements. Green, DiTV3a ORFs; blue, host genes with retrotransposon-related genes labeled (endonuclease-reverse transcriptase, activating signal cointegrator 1 complex subunit, GATA zinc finger domain-containing protein 14-like); gray, hypothetical proteins.

Some DiTV3a ORFs were associated with putative retrotransposon elements. Genomic fragments comprised of DiTV3a genes and retrotransposon-related genes were observed in both SCR and NCR samples (Figure 2), but integrations varied between contigs derived from SCR and NCR. For instance, DiTV3a_F14_ORF2 and ORF3 were assembled with a DNA fragment of 7219 bp, wherein an ORF encoding an endonuclease-reverse transcriptase was predicted in the SCR contig. The other putative ORFs in DiTV3a_F14 “hit” three uncharacterized protein coding loci in the WCR genome assembly, LOC114344791, LOC114341432 and LOC114348326 (Figure 2). Similarly, an NCR-derived 8295 bp contig was assembled containing DiTV3a_F4_ORF2 and ORF3 genes together with genes encoding a retrovirus-related activating signal cointegrator 1 complex subunit (Pol polyprotein family) from transposon 412-like protein and a GATA zinc finger domain-containing protein 14-like protein (Figure 2).

### 3.6. Toursvirus Proteins Are More Similar to Those of Iridoviruses Than Ascoviruses

**Figure 3:**
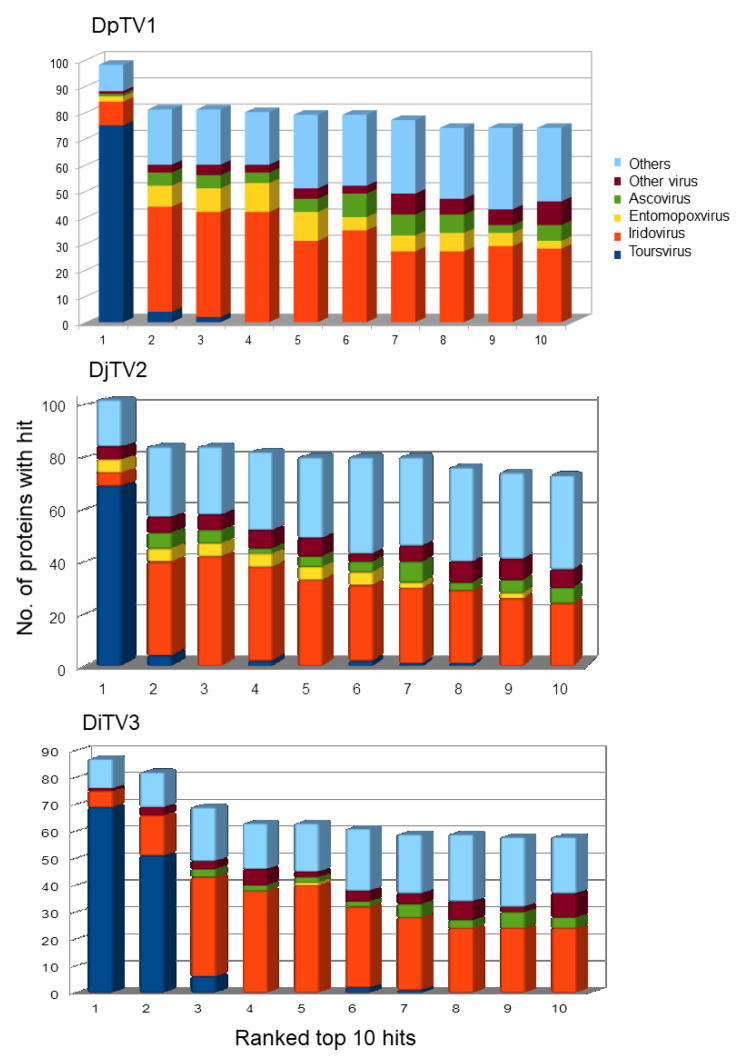
Similarity of toursvirus proteins to proteins from other virus species. The virus group associated with the top 10 non-redundant hits from 1 to 10 from BLASTp analysis against the NCBI nr database are shown for putative ORFs of DpTV1, DjTV2 and DiTV3a. The numbers of proteins with similarity to the different virus groups or to other proteins are shown.

Our BLASTp searches resulted in identification of 90 putative DiTV3a gene translations that matched known viral proteins (Table 3). Seventy-one DiTV3a genes were similar to known toursviral genes. There were also 19 ORFs that hit the genes of other viruses (mainly iridoviruses) or proteins of non-viral origin. Surprisingly, none of the top hits were from ascoviral genes. Assessment of similarity among the 119 DpTV1a proteins, 141 DjTV2a proteins, 120 DiTV3a proteins and those of related DNA viruses showed that the majority (~70%) of the BLASTp top hits to DpTV1a proteins were to proteins of DjTV2a, and vice versa (Figure 3). The majority of the top 1 and top 2 hits from queried DiTV3a proteins were to either DpTV1a or DjTV2a, and the top 3 to top 10 hit viral species were iridoviruses (Table S3). Less than 10% of the top 1 to top 10 hits for DiTV3a were to ascoviruses. While entomopoxviruses were frequent among the “hits”, almost all of these were to *bro* genes in DpTV1a or DjTV2a, which were not identified in DiTV3a. Only one entomopoxvirus hit was observed in the top 10 hit species of DiTV3a. A few DpTV1a proteins hit ichnovirus proteins. Hits to marseilleviruses were frequently observed, and protein sequences of *Pithovirus*, a group of giant DNA viruses, were frequently hit by toursviruses in the BLASTp search. Interestingly, at least 25% of the top 2–10 hits of the toursviral proteins were from bacteria and other non-viral organisms, demonstrating the diversity in the composition of toursviral genomes. Taken together, our analysis of similarity among all putative proteins encoded by the three toursviruses showed greatest DiTV3 protein similarity to iridovirus proteins, with relatively little similarity to *Ascovirus* proteins.

### 3.7. Phylogenetic Analyses Indicate That Toursviruses form a Distinct Clade

**Figure 4:**
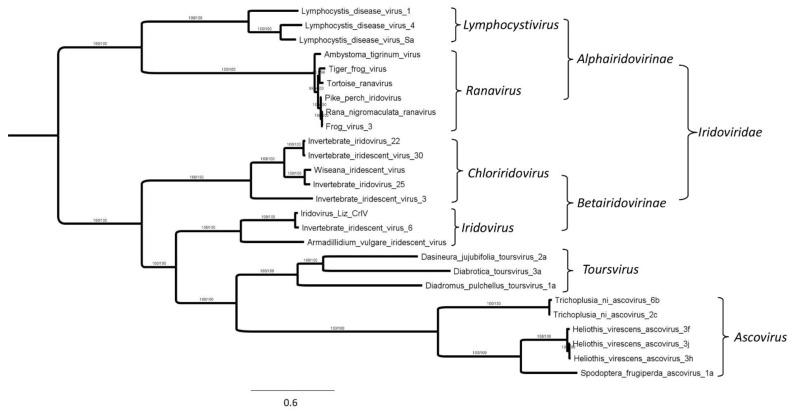
Phylogenetic tree based on the concatenated sequences of 45 proteins encoded by 26 toursviruses, iridoviruses and ascoviruses. Protein sequences were selected based on BLASTp results (*E* value ≤ 0.001) and downloaded from the NCBI protein database. Methods for phylogenetic tree construction are as described in materials and methods. Bootstrap values (percent) are indicated. Virus groups are shown at right. Corresponding protein accession numbers for each virus are provided in Table S1.

To assess the evolutionary relationships among ascoviruses, toursviruses, and iridoviruses, a phylogenetic tree was generated based on the concatenated protein sequences in silico translated from 45 genes encoded by 26 viruses. The sequences used were derived from four genera of *Iridoviridae*, specifically *Lymphocystivirus*, *Ranavirus* (*Alphairidovirinae*), *Chloriridovirus,* and *Iridovirus* (*Betairidovirinae*). Sequences derived from *Meglocytivirus* (*Alphairidovirnae*) and *Decapodiridovirus* (*Betairidovirinae*) were not included in the phylogenetic analysis due to low sequence similarity to those of toursviruses. The tree predicts clustering of toursviruses into a distinct clade (Figure 4). The tree supports the premise that toursviruses are phylogenetically closer to members of *Iridovirus* than to those of *Ascovirus* (Figure 4).

## 4. Discussion

We previously reported two novel DNA viruses (nudiviruses) identified from genome sequence data of SCR and WCR [34]. Here we identify the third DNA virus sequence from *Diabrotica* spp., which is from a novel toursvirus in SCR and NCR. An estimated 90% of the DiTV3a genomic DNA was recovered following assembly of short read sequencing data from the host genomes. However, relatively short DNA fragments were assembled with many gaps, and further work will be required to generate the complete genome sequence. DiTV3a sequences isolated from SCR and NCR were almost identical, indicating these two isolates may be derived from closely related lineages of the same virus. One hundred and twenty putative ORFs were predicted from the 26 DiTV3a genomic fragments. Sequences of DiTV3a were found in SCR and NCR, but not from the previously analyzed WCR genomic sequences [34]. DiTV3 is the first toursvirus identified in Coleoptera.

### 4.1. Genome Assembly

The DiTV3a sequences were assembled into twenty-six fragments, with the longest less than 20 kbp. It is not clear why longer fragments of DiTV3a were not assembled. Technical parameters that could account for this include 100 bp single end reads being less tractable for assembly than paired end reads, repetitive regions within the genome hindering assembly, and assembly parameters. Some genes commonly found in other ascoviruses (e.g., *ABC transport system permease* and *bro*) were not identified from the assembled DiTV3a fragments, suggesting either that some sequence regions of the DiTV3 genome were not assembled, or that these genes were absent from this virus. One possible explanation is that viral sequence coverage was insufficient for recovery of sequence reads in all regions of the DiTV3 genome. We previously discovered a near full length nudivirus genome sequence (DuhNV) from the same SCR DNA sample. The average base coverage of DuhNV was less than 19-fold [34], ~3-fold less than that of DiTV3a, suggesting that the number of reads derived from DiTV3a should be sufficient to generate longer fragments. Therefore, additional factors likely account for the poor DiTV3a genome assembly. In contrast to DiTV3a sequences which were associated with retrotransposon elements in both SCR and NCR, no retroviral elements were associated with the DuhNV sequences. It is conceivable that the DiTV3a genome sequence has been disrupted by retrotransposon activity, potentially resulting in our inability to identify genes such as *ABC transport system permease* and *bro* using the BLAST parameters employed.

Both *ABC transport system permease* and *bro* are multi-gene families, members of which are commonly found in toursviruses and ascoviruses. Based on sequences deposited in NCBI, 2 to 6 ABC transport system permease genes are found in toursviruses and ascoviruses, except for the *Spodoptera frugiperda ascovirus 1a*, SfAV 1a. Three to 25 bro genes are found in toursviruses and ascoviruses. DpTV1 and DjTV2 each encode two *ABC-type transport system permease* genes, and 9 and 5 copies of *bro*, respectively. It is unknown why these two genes were not identified from the recovered DiTV3a genome sequence. To address whether partial sequences of these genes are present in the short contigs, we translated the SCR and NCR contigs with the six-frame translation option. The resulting protein sequences were aligned by BLASTp using protein sequences encoded by DpTV1 and DjTV2 as reference. No sequences encoding potential ABC-type transport system permease or Bro proteins were detected. Therefore, it is unlikely that these sequences were missed due to poor DiTV3a genome assembly. It is possible that DiTV3a either does not encode ABC-type transport system permease related proteins or Bro proteins, or that these genes have been disrupted by transposon activity. It is notable in this context that *Spodoptera frugiperda ascovirus 1a* lacks the permease gene [26].

### 4.2. Sequence Integration

Differential association of transposon-related sequences with DiTV3a sequences recovered from SCR and NCR indicates structural variation between the two isolates, even though the virus-derived sequences are highly similar. As the three putative WCR genes in Figure 2 are not on the same scaffold in the WCR genome assembly, these host genes could have been acquired and integrated into the viral genome. Such integration of host sequences into viral genomes has been described previously in large DNA viruses of insects (*Baculoviridae*) [47,48].

The integration of viral genomes into host genomes is not uncommon for DNA viruses, including a member of *Iridoviridae*, Frog virus 3 [49]. However, the underlying mechanisms of integration events are poorly understood [49,50]. It is unclear whether the genome sequences of DiTV3a were integrated into the genomes of SCR or NCR in the samples used for this work. However, previous analysis of SCR transcriptomes [33] did not reveal toursvirus RNAs, which would be expected for intact DiTV3a ORFs if the virus was integrated into the host genome.

### 4.3. Evolutionary Relationships

A close relationship between *Ascoviridae* and *Iridoviridae* was previously observed by phylogenetic analysis of their DNA polymerases [51]. DpTV1 was also shown at the evolutionary intersection of iridoviruses, ascoviruses, and ichnoviruses [25]. Currently, *Ascoviridae*, *Iridoviridae*, and *Marseilleviridae* are assigned to the Order *Pimascovirales* (in Realm: *Varidanviria*, Kingdom: *Bamfordvirae*, and Phylum: *Nucleocytoviricoda*) (https://talk.ictvonline.org/taxonomy; accessed on 27 January 2021). At present, DpTV1a is the only toursvirus recognized by the International Committee on Taxonomy of Viruses (ICTV). The recently reported DjTV2a [26] and DiTV3a presented in this manuscript are two new members of the *Toursvirus* genus.

The comprehensive phylogenetic analysis based on 45 viral protein sequences showed greater similarity of toursvirus proteins to those of iridoviruses, than to those of ascoviruses (Figure 4). This result is consistent with the high numbers of iridovirus hits on BLASTp analysis of toursvirus proteins, with relatively few from ascovirus proteins (Figure 3). Indeed, phylogenetic analyses for each of 28 core genes for the first identified toursvirus (DpTV1) showed that 17 core genes supported the hypothesis that DpTV1 is more closely related to iridoviruses and belongs to a clade distinct from *Ascovirus* [25].

Based on this analysis, *Toursvirus* and *Ascovirus* should not be taxonomically grouped together in *Ascoviridae*. The extensive sequence divergence of the *Ascovirus* genus from the *Toursvirus* genus since their evolution from a common ancestor forms the basis for this recommendation. We propose that the genus *Toursvirus* be separated from *Ascoviridae,* and a new family *Toursviridae* be created within the order *Pimascovirales*. *Pimascovirales* would then contain four families: *Ascoviridae*, *Iridoviridae*, *Marseilleviridaes*, and *Toursviridae*.

## Figures and Tables

**Table 1:** Characteristics of ascovirus genomes.

Virus	Abbr.	% G+C	Accession	Length (bp)	Putative CDS	Host	Ref
**Genus *Ascovirus***							
*Spodoptera frugiperda ascovirus 1a*	SfAV1a	49.26	NC_008361.1	156922	123	*Spodoptera frugiperda*	[19]
*Trichoplusia ni ascovirus 2c*	TNAV2c	35.24	NC_008518.1	174059	165		[20]
*Heliothis virescens ascovirus 3e*	HVAV3e	45.88	NC_009233.1	186262	178		[21]
*Heliothis virescens ascovirus 3f*	HVAV3f	46	NC_044938.1	198157	190	*Helicoverpa zea*	
*Heliothis virescens ascovirus 3g*	TnAV3g	45.85	JX491653.1	199721	194	*Spodoptera exigua*	[22]
*Heliothis virescens ascovirus 3h*	HvAV3h	45.5	KU170628.1	190519	185	*Spodoptera exigua*	[23]
*Heliothis virescens ascovirus 3i*	HvAV3i	45.42	MF781070.1	185650	181	*Spodoptera frugiperda*	[24]
*Heliothis virescens ascovirus 3j*	HvAV3j	45.62	LC332918.1	191718	189	*Spodoptera litura*	
*Trichoplusia ni ascovirus 6b*	TnAV6b	35.43	KY434117.1	185664	178	*Helicoverpa zea*	
**Genus *Toursvirus***							
Diadromus pulchellus toursvirus 1a *	DpTV1a	49.16	NC_011335.1	119343	119	*Diadromus puchellus*	[25]
Dasineura jujubifolia toursvirus 2a	DjTV2a	45.97	MK867691.1	142600	141	*Dasineura jujubifolia*	[26]

* Previously named *Diadromus pulchellus ascovirus 4a* [27]. Virus names in italics are recognized by the International Committee on Virus Taxonomy.

**Table 2:** Sequence coverage and nucleotide content of DiTV3a genome fragments (F1 to F26).

Fragment	Length (bp)	% G+C	No. Putative CDS	Total Reads Mapped		Average Base Coverage	
				SCR	NCR	SCR	NCR
F1	9099	30.02	9	4775	2333	52.48	25.64
F2	19,537	29.5	22	10,479	2763	53.64	14.14
F3	10,800	29.88	12	5553	1494	51.42	13.83
F4	9493	28.68	13	4941	1670	52.05	17.59
F5	6898	31.01	4	3533	880	51.22	12.76
F6	5330	30.17	5	2746	733	51.52	13.75
F7	4680	28.44	7	2022	636	43.21	13.59
F8	4514	30.24	4	2275	590	50.40	13.07
F9	4062	30.66	4	2048	800	50.42	19.69
F10	3943	30.97	2	2057	520	52.17	13.19
F11	3641	30.82	5	1794	655	49.27	17.99
F12	2725	27.78	4	1390	348	51.01	12.77
F13	2590	28.3	4	1346	346	51.97	13.36
F14	2473	26.97	3	1252	440	50.63	17.79
F15	2445	30.35	1	1234	274	50.47	11.21
F16	2273	30.23	2	1122	344	49.36	15.13
F17	1970	28.63	3	994	196	50.46	9.95
F18	1606	27.21	3	813	178	50.62	11.08
F19	1586	31.97	2	725	164	45.71	10.34
F20	1487	31.07	2	698	182	46.94	12.24
F21	1327	24.86	2	714	220	53.81	16.58
F22	1135	29.46	2	623	156	54.89	13.74
F23	1305	32.43	1	604	274	46.28	21.00
F24	1270	29.53	1	600	316	47.24	24.88
F25	1135	29.53	2	510	242	44.93	21.32
F26	826	40.56	1	357	266	43.22	32.20
	108,150	29.9719231	120	55,205	17,020	51.94	15.74

**Table 3:** Putative genes of DiTV3a per genome fragment.

ORF	Length (aa)	Mr (kDa)	Gene	Similar ORFs	Functional Category †
F1_ORF1	166 *	18.7	hypothetical protein	IIV6_404L (Invertebrate iridovirus 6)	
F1_ORF2	154	18.5	hypothetical protein	N/A	
F1_ORF3	386	45	ribonucleoside-diphosphate reductase subunit M2	DjTV_ORF44/AV955_gp066	
F1_ORF4	124	15.4	hypothetical protein	N/A	
F1_ORF5	181	21.1	casein kinase 1, delta	none from viruses	
**F1_ORF6**	941	109.1	DNA polymerase	DjTV_ORF1/AV955_gp001	1
F1_ORF7	59	6.8	hypothetical protein	N/A	
F1_ORF8	109	12.8	mobilome: prophages, transposons	phage anti-repressor protein	
F1_ORF9	580	66.5	hypothetical protein	DjTV_ORF30/AV955_gp054	
F2_ORF1	85	9.9	hypothetical protein	N/A	
*F2_ORF2*	774	90.5	lipopolysaccharide-modifying enzyme	DjTV_ORF114/AV955_gp105	7
F2_ORF3	151	17.5	hypothetical protein	DjTV_ORF2/AV955_gp039	
F2_ORF4	197	22.4	hypothetical protein	DjTV_ORF98/AV955_gp024	
F2_ORF5	280	33.9	hypothetical protein (hit CDD pfam08793, 2c_adapt [cl07414], PTZ00449 [cl33186])	none from viruses	
F2_ORF6	136	15.6	hypothetical protein	DjTV_ORF100	
F2_ORF7	800	93.7	hypothetical protein	DjTV_ORF57/AV955_gp094	
**F2_ORF8**	158	18.9	putative zinc-finger DNA binding protein	DjTV_ORF129/AV955_gp108	7
F2_ORF9	195	21.9	hypothetical protein	N/A	
**F2_ORF10**	871	101.1	DEAD-like helicase	DjTV_ORF81/AV955_gp020	1
F2_ORF11	226	26.9	UMP-CMP kinase 2, mitochondrial-like (thymidylate kinase)	none from viruses	
F2_ORF12	196	22.5	thymidylate kinase	ORF of *Fowlpox virus*	
F2_ORF13	181	20.5	hypothetic protein histone-lysine N-methyltransferase 2C-like	none from viruses	
F2_ORF14	117	13.3	hypothetical protein	N/A	
F2_ORF15	459	52.6	DNA ligase	R303 (*Acanthamoeba* *polyphaga mimivirus*)	
F2_ORF16	50	6.1	hypothetical protein	N/A	
*F2_ORF17*	278	32	hypothetical protein	DjTV_ORF90/AV955_gp035	7
*F2_ORF18*	250	29.4	acetyltransferase	DjTV_ORF115/AV955_gp081	5
F2_ORF19	108	13.2	hypothetical protein	N/A	
F2_ORF20	146	17	acyl-CoA-binding protein domain containing protein	none from viruses	
F2_ORF21	91	10.9	hypothetical protein	N/A	
F2_ORF22	164	19.2	hypothetical protein	DjTV_ORF58/AV955_gp095	
F3_ORF1	135	16.1	hypothetical protein	DjTV_ORF19/AV955_gp071	
**F3_ORF2**	328	37.8	DNA repair exonuclease	DjTV_ORF18/AV955_gp026	1
F3_ORF3	87	10.4	hypothetical protein	N/A	
F3_ORF4	240	28.4	hypothetical protein	DjTV_ORF28/AV955_gp013	
**F3_ORF5**	1031	122.2	dynein-like beta chain protein	DjTV_ORF94/AV955_gp085	7
F3_ORF6	464	54.6	hypothetical protein	DjTV_ORF94/AV955_gp079	
**F3_ORF7**	263	31.1	RNaseIII	DjTV_ORF22/AV955_gp003	2
F3_ORF8	349	41.3	flap structure-specific endonuclease	DH26_gp060 (Anopheles minimus irodovirus)	1
F3_ORF9	162	19.4	hypothetical protein	N/A	
F3_ORF10	67	7.9	hypothetical protein	N/A	
F3_ORF11	71	7.9	hypothetical protein	N/A	
F3_ORF12	60 *	7.1	hypothetical protein	N/A	
F4_ORF1	117	13.6	hypothetical protein	DjTV_ORF107/AV955_gp045	
F4_ORF2	130	15.5	hypothetical protein	DjTV_ORF118/AV955_gp072	
**F4_ORF3**	345	41.4	DNA binding/packing protein	DjTV_ORF99/AV955_gp103	3
F4_ORF4	123	14.9	hypothetical protein	N/A	
**F4_ORF5**	455	51.8	major capsid protein	DjTV_ORF83/AV955_gp019	3
F4_ORF6	132	15.5	thioredoxin-like protein	DjTV_ORF116/AV955_gp104	
**F4_ORF7**	365	43.3	immediate early protein ICP-46	DjTV_ORF97/AV955_gp043	7
**F4_ORF8**	104	12.4	yabby-like transcription factor	DjTV_ORF110/AV_955_gp022	2
F4_ORF9	207	23.6	hypothetical protein	DjTV_ORF92/AV955_gp023	
F4_ORF10	146	17.3	putative RING finger protein	IIV22_063R (Invertebrate iridovirus 22)	
F4_ORF11	98	11.4	hypothetical protein	putative protein 4 (Dougjudy virga-like virus)	
*F4_ORF12*	239	28.5	casein kinase 1-like protein 5/major virion DNA-binding protein	DjTV_ORF54/AV955_gp115	3
F4_ORF13	277	31.4	hypothetical protein	N/A	
**F5_ORF1**	1029	116.2	DdRp II	DjTV_ORF111/AV955_gp073	2
F5_ORF2	60	7.2	hypothetical protein	N/A	
F5_ORF3	650	76.1	hypothetical protein	DjTV_ORF105	
F5_ORF4	287	33.4	hypothetical protein	N/A	
F6_ORF1	66 *	8.1	hypothetical protein	N/A	
F6_ORF2	143	16.2	hypothetical protein	N/A	
F6_ORF3	982	115	RHS repeat protein	none from viruses	
F6_ORF4	122	14.5	hypothetical protein	DjTV_ORF69	
F6_ORF5	279	32.7	hypothetical protein	DjTV_ORF46/AV955_gp109	
F7_ORF1	50	5.8	hypothetical protein	N/A	
F7_ORF2	61	7	hypothetical protein	N/A	
F7_ORF3	176	21.1	uyr/REP helicase	DjTV_ORF89/AV955_gp028	
**F7_ORF4**	622	72.8	ATPase	DjTV_ORF4/AV955_gp033	1
F7_ORF5	123	14.3	hypothetical protein	N/A	
F7_ORF6	170	19.5	hydrolase, NUDIX family	DjTV_ORF83/AV955_gp005	
F7_ORF7	81 *	9.7	putative RING finger protein	MIV027R (Invertebrate iridescent virus 3)	7
**F8_ORF1**	836	97.6	ATPase	DjTV_ORF63/AV955_gp093	1
F8_ORF2	84	9.6	hypothetical protein	DjTV_ORF91/AV955_gp030	
**F8_ORF3**	189	22.6	thymidine kinase	DjTV_ORF12/AV955_gp055	1
*F8_ORF4*	224	27.2	fatty acids protein	DjTV_ORF127/AV955_gp015	5
**F9_ORF1**	408	46.9	RNA polymerase II	DjTV_ORF10/AV955_gp070	2
F9_ORF2	126	14.6	thiredoxin-like	DjTV_ORF128/AV955_gp044	
*F9_ORF3*	290	33.8	myristylated membrane protein-like protein	DjTV_ORF40/AV955_gp065	7
F9_ORF4	353 *	40	dUTP diphosphatase	none from viruses	
**F10_ORF1**	877 *	116.2	DdRp	DjTV_ORF7/AV955_gp089	2
F10_ORF2	356	42.4	hypothetical protein	IIV31_072L (Armadillidium vulgare iridescent virus)	
*F11_ORF1*	254 *	29.6	myristylated membrane protein-like protein	DjTV_ORF40/AV955_gp065	5
F11_ORF2	126	14.6	thiredoxin-like	DjTV_ORF128/AV955_gp044	
F11_ORF3	289	33.3	thymidylate synthase	none from viruses	
F11_ORF4	216	25	hypothetical protein	DjTV_ORF77/AV955_gp031	
F11_ORF5	133	15.6	transcription elongation factor S-II	DjTV_ORF78/AV955_gp082	
**F12_ORF1**	152	18.5	hypothetical protein	DjTV_ORF26/AV955_gp010	
**F12_ORF2**	519	60.7	hypothetical protein	DjTV_ORF25/AV955_gp036	4
F12_ORF3	57	6.5	hypothetical protein	N/A	
F12_ORF4	83 *	9.3	hypothetical protein	N/A	
F13_ORF1	79	9.5	hypothetical protein	N/A	
F13_ORF2	104	11.9	IIV22A_144R-like	IIV22A_144R (Invertebrate iridescent virus 22)	
*F13_ORF3*	206	24.3	zinc-dependent metalloprotease	DjTV_ORF119/AV955_gp029	7
**F13_ORF4**	360	40.8	major virion DNA-binding protein	DjTV_ORF98/AV955_gp008	3
F14_ORF1	117	136	DNA-directed RNA polymerases I, II, and III	DjTV_ORF66/AV955_gp058	
F14_ORF2	202	23.5	hypothetical protein	DjTV_ORF103/AV955_gp051	
**F14_ORF3**	264	31.2	hypothetical protein	DjTV_ORF59/AV955_gp009	
*F15_ORF1*	728	83.9	serine/threonine protein kinase	DjTV_ORF55/AV955_gp046	4
F16_ORF1	269	31	hypothetical protein	DjTV_ORF87/AV955_gp025	
**F16_ORF2**	384	44.1	hypothetical protein	DjTV_ORF86/AV955_gp064	
*F17_ORF1*	260	29.8	patatin-like phospholipase	DjTV_ORF140/AV955_gp087	5
F17_ORF2	118	13.4	hypothetical protein	AV955_gp107	
**F17_ORF3**	174	20.4	hypothetical protein	DjTV_ORF9/AV955_gp116	
F18_ORF1	95 *	11.4	hypothetical protein	N/A	
F18_ORF2	214	25.9	hypothetical protein	DjTV_ORF121/AV955_gp060	
F18_ORF3	140	16.9	hypothetical protein	N/A	
F19_ORF1	79 *	9.3	hypothetical protein	N/A	
**F19_ORF2**	445	49	lipid membrane protein	DjTV_ORF61/AV955_gp040	
**F20_ORF1**	348	39.9	putative myristylated membrane protein	AV955_gp065	7
*F20_ORF2*	62 *	6.9	hypothetical protein	DjTV_ORF67/AV955_gp063	
**F21_ORF1**	170	20.5	CDT phosphatase transcription factor	DjTV_ORF81/AV955_gp117	2
F21_ORF2	148	17.2	hypothetical protein	DjTV_ORF82/AV955_gp097	
**F22_ORF1**	104	12.3	sulfhydry1 oxidase Erv1 like protein	DjTV_ORF62/AV955_gp041	
**F22_ORF2**	257	30	ATPase 3	DjTV_ORF137/AV955_gp086	
**F23_ORF1**	358	40.9	cathepsin B	DjTV_ORF50/AV955_gp048	6
F24_ORF1	406	47.8	hypothetical protein	DjTV_ORF72/AV955_gp096	
**F25_ORF1**	193 *	22.7	iap-3	DjTV_ORF108/AV955_gp007	6
F25_ORF2	73	8.5	hypothetical protein	N/A	
F26_ORF1	234	27.1	hypothetical protein	MIV075R (Invertebrate iridescent virus 3)	

Bold, genes shared by ascoviruses [25]; Italic, identified by Wang et al., [26]; * partial sequences; † Gene function: 1, DNA replication and repair; 2, transcription; 3 Structural protein; 4, protein modification; 5, lipid metabolism; 6, apoptosis; 7, other.

## Data Availability

Raw sequence data used for this analysis are available as indicated in Section 2.2 of the manuscript. DiTV3a sequence is provided as a text file in Supplementary Materials.

## Supplementary Materials

The following Supplementary Materials are available online at https://www.mdpi.com/article/10.3390/v14020397/s1. Table S1. Accession numbers for viral proteins used for construction of the phylogenetic tree. Table S2. Summary of DiTV3a sequence analysis. Table S3. Species associated with the 10 most similar proteins to putative proteins encoded by DpTV1, DjPV2 and DiTV3a as depicted in Figure 3. DiTV3a sequence, text file containing sequences of DiTV3 identified in this study. Figure S1. DiTV3a genomic fragments (28) isolated from the southern corn rootworm. Figure S2. DiTV3a genomic fragments (42) isolated from the northern corn rootworm. File S1. DiTV3a sequence.
