# Supplementary material for: Sequences Encoding a Novel Toursvirus Identified from Southern and Northern Corn Rootworms (Coleoptera: Chrysomelidae)
Source: Viruses. 2022 Feb 15;14(2):397. doi: 10.3390/v14020397 (PMC8879594; doi:10.3390/v14020397)
Supplement: Supplementary file 1 [file viruses-14-00397-s001.zip › Supplementary Materials Revised/SupplementaryFigures.pdf]

SUPPLEMENTARY INFORMATION

Sequences encoding a novel toursvirus identified from southern and northern corn rootworms (Coleoptera: Chrysomelidae)

Sijun Liu [1], Thomas W. Sappington [2], Brad S. Coates [2], Bryony C. Bonning [3]

Maps of DiTV3a genomic fragments isolated from SCR (Figure S1) and NCR (Figure S2). For both figures, Arrows indicate ORFs and ORF orientation. ORFs that hit toursvirus genes (DjTV2a/DpTV1a) are indicated.

Figure S1. DiTV3a genomic fragments (28) isolated from the southern corn rootworm.

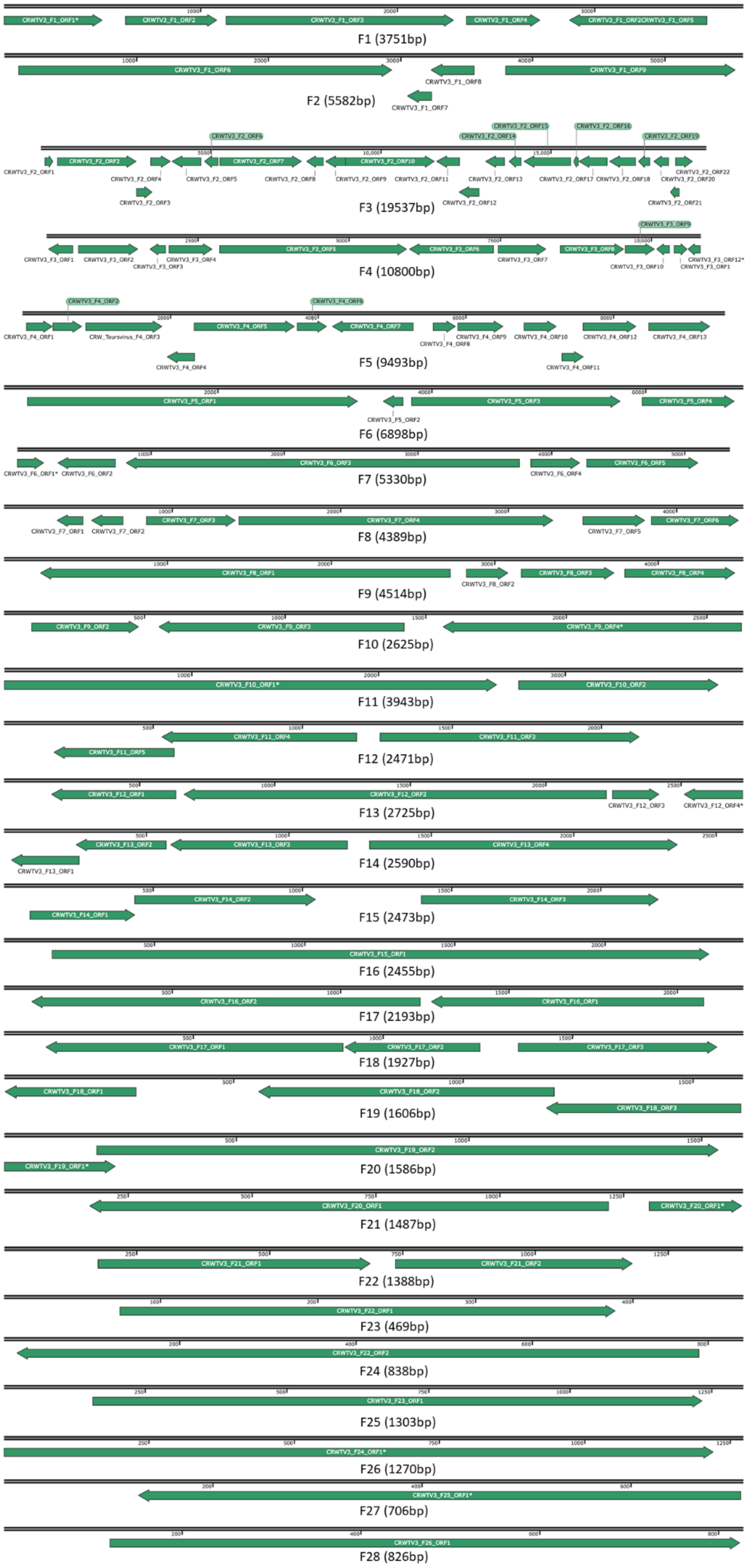

Figure S2. DiTV3a genomic fragments (42) isolated from the northern corn rootworm

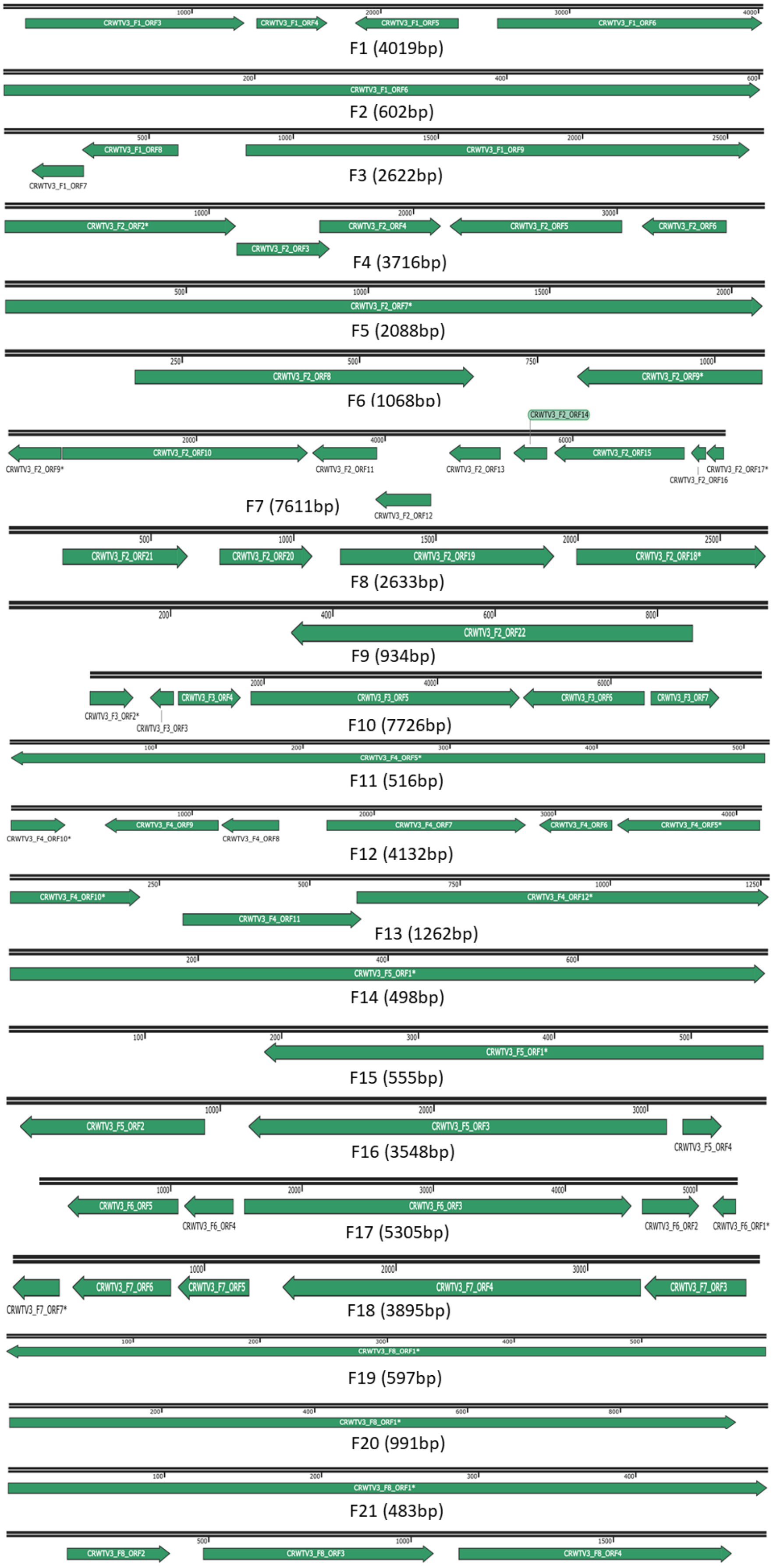

Figure S2 ctd.

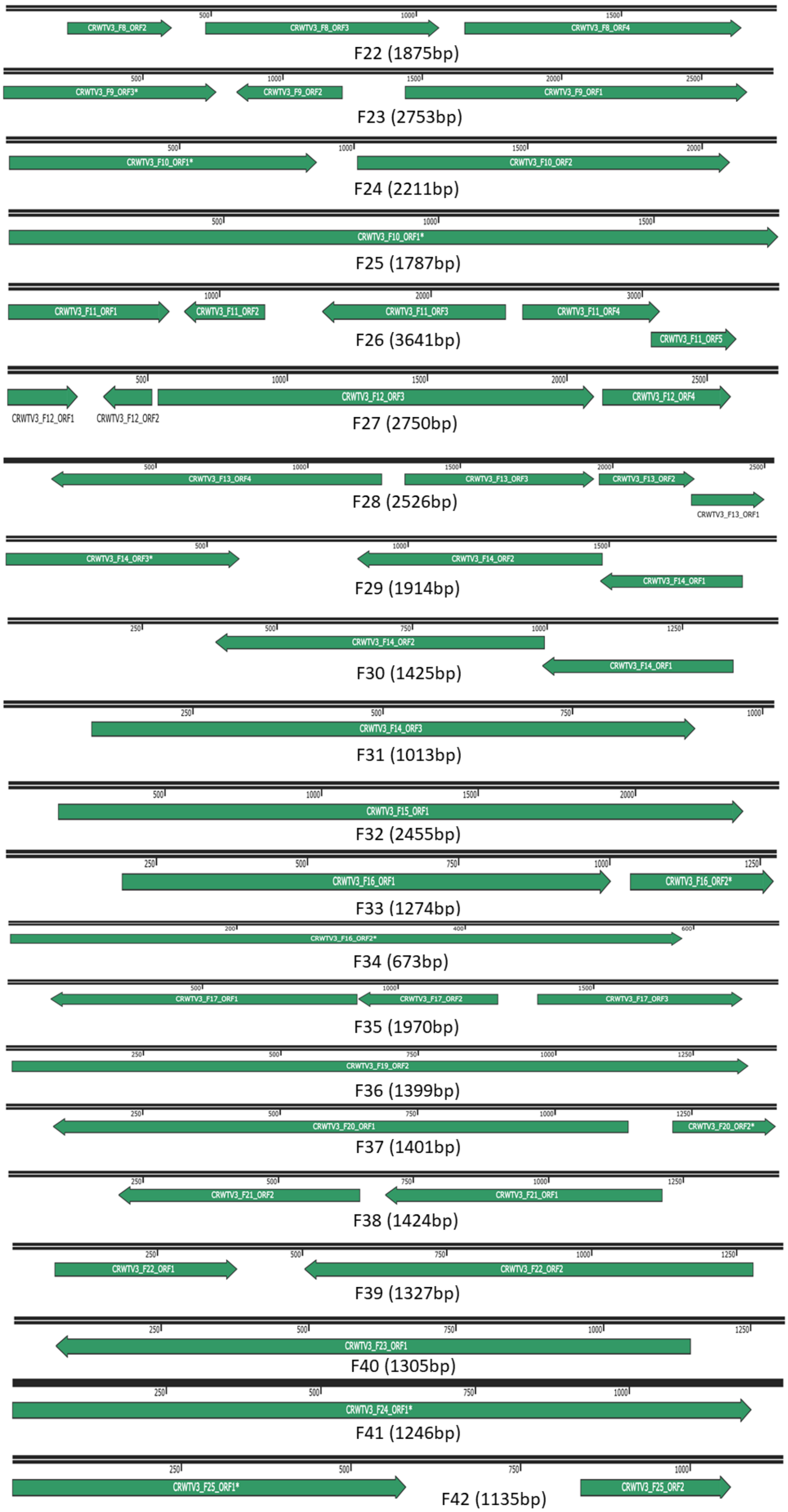
CRWTV3_F8_ORF2
CRWTV3_F8_ORF3
CRWTV3_F8_ORF4
F22 (1875bp)
CRWTV3_F9_ORF3*
CRWTV3_F9_ORF2
CRWTV3_F9_ORF1
F23 (2753bp)
CRWTV3_F10_ORF1*
CRWTV3_F10_ORF2
F24 (2211bp)
CRWTV3_F10_ORF1*
F25 (1787bp)
CRWTV3_F11_ORF1
CRWTV3_F11_ORF2
CRWTV3_F11_ORF3
CRWTV3_F11_ORF4
F26 (3641bp)
CRWTV3_F11_ORF5
CRWTV3_F12_ORF3
CRWTV3_F12_ORF4
CRWTV3_F12_ORF1
CRWTV3_F12_ORF2
F27 (2750bp)
CRWTV3_F13_ORF4
CRWTV3_F13_ORF3
CRWTV3_F13_ORF2
F28 (2526bp)
CRWTV3_F13_ORF1
CRWTV3_F14_ORF3*
CRWTV3_F14_ORF2
CRWTV3_F14_ORF1
F29 (1914bp)
CRWTV3_F14_ORF2
CRWTV3_F14_ORF1
F30 (1425bp)
CRWTV3_F14_ORF3
F31 (1013bp)
CRWTV3_F15_ORF1
F32 (2455bp)
CRWTV3_F16_ORF1
CRWTV3_F16_ORF2*
F33 (1274bp)
CRWTV3_F16_ORF2*
F34 (673bp)
CRWTV3_F17_ORF1
CRWTV3_F17_ORF2
CRWTV3_F17_ORF3
F35 (1970bp)
CRWTV3_F19_ORF2
F36 (1399bp)
CRWTV3_F20_ORF1
CRWTV3_F20_ORF2*
F37 (1401bp)
CRWTV3_F21_ORF2
CRWTV3_F21_ORF1
F38 (1424bp)
CRWTV3_F22_ORF1
CRWTV3_F22_ORF2
F39 (1327bp)
CRWTV3_F23_ORF1
F40 (1305bp)
CRWTV3_F24_ORF1*
F41 (1246bp)
CRWTV3_F25_ORF1*
CRWTV3_F25_ORF2
F42 (1135bp)
